# The impact of a healthy lifestyle on Disability-Adjusted Life Years: a prospective cohort study

**DOI:** 10.1186/s12916-015-0287-6

**Published:** 2015-02-27

**Authors:** Anne M May, Ellen A Struijk, Heidi P Fransen, N Charlotte Onland-Moret, G Ardine de Wit, Jolanda MA Boer, Yvonne T van der Schouw, Jeljer Hoekstra, H Bas Bueno-de-Mesquita, Petra HM Peeters, Joline WJ Beulens

**Affiliations:** Julius Center for Health Sciences and Primary Care, University Medical Centre Utrecht, Huispost Str. 6.131, PO Box 85500, Utrecht, 3508 GA The Netherlands; National Institute for Public Health and the Environment (RIVM), PO Box 1, Bilthoven, 3720 BA The Netherlands; Department of Gastroenterology and Hepatology, University Medical Centre Utrecht, PO Box 85500, Utrecht, 3508 GA The Netherlands; School of Public Health, Imperial College London, London, W2 1PG UK

**Keywords:** BMI, Disability-Adjusted Life Years (DALY), Disease burden, EPIC-NL, Lifestyle, Mediterranean diet, Physical activity, Smoking

## Abstract

**Background:**

The association between single health behaviours and incidence of and premature mortality from major chronic diseases, including myocardial infarction, stroke, diabetes mellitus, and cancer, has been demonstrated thoroughly. However, the association of several healthy behaviours with Disability-Adjusted Life Years (DALYs), which is a measure for total health combining Years Lost due to Disability and the Years of Life Lost due to premature mortality, has not been studied yet.

**Methods:**

A prospective cohort study was conducted among 33,066 healthy men and women aged 20 to 70 years recruited into the EPIC-NL study during 1993 to 1997. Participants’ smoking status, BMI, physical activity, and adherence to a Mediterranean-style diet (excluding alcohol) were investigated separately and combined into a simple health behaviour score ranging from 0 to 4. Participants were followed until the end of 2007 for occurrence of and mortality from the most important chronic diseases. The association between lifestyle (separate lifestyle factors and a simple health behaviour score) and DALYs were adjusted for relevant confounders.

**Results:**

After a median follow-up of 12.4 years, 6,647 disease incidences and 1,482 deaths were documented. Non-smoking, low BMI (BMI <25), being physically active, and adherence to a Mediterranean diet were all associated with a significantly lower disease burden. Persons adhering to all four healthy lifestyle characteristics lived a minimum of 2 years longer in good health (DALYs: −2.13; 95% CI: −2.65 to −1.62) than persons with none. Due to our non-extinct cohort, the total number of DALYs, and consequently the estimates, is underestimated. Therefore, true lifetime health benefits of a healthy lifestyle will be even larger.

**Conclusions:**

Non-smoking, a low BMI, being physically active, and adherence to a Mediterranean diet were associated with a lower disease burden. Each additional healthy lifestyle factor contributed to a longer life in good health.

**Electronic supplementary material:**

The online version of this article (doi:10.1186/s12916-015-0287-6) contains supplementary material, which is available to authorized users.

## Background

Global life expectancy has increased considerably in the last decades, for both men and women [1]. In the Netherlands, life expectancy is estimated to further increase from 73.1 and 82.8 years in 2012 to 85.7 and 88.5 years in 2050 for men and women, respectively [2]. Nevertheless, people who live longer are not necessarily in good health [3]. Therefore, it is increasingly important to investigate potentially modifiable factors that are related to living longer in good health. Unhealthy behaviours, such as being physically inactive and smoking, are leading contributors to morbidity and mortality [3,4]. Moreover, recent studies showed that people who combine more health behaviours, i.e., who are physically active, non-smoking, and who have a normal body weight and eat healthily, have a reduced risk of major chronic diseases including myocardial infarction, stroke, diabetes mellitus, and cancer. These behaviours were also related to lower mortality [5-19].

The use of Disability-Adjusted Life Years (DALYs), a summary measure of health combining both morbidity and mortality, as an outcome instead of specific disease incidence or mortality, enables us to investigate the relationship of health behaviours with total instead of a specific disease burden [20]. To date, DALYs are mainly calculated on a population level based on statistical data of disease incidence and mortality. To relate risk factors to the estimated DALYs, effect sizes for each risk factor based on available observational or intervention studies are used. Using this approach, the Global Burden of Disease study 2010 recently showed that unhealthy dietary components and physical inactivity, tobacco smoking, and high body mass index (BMI) were responsible for 10.0%, 6.3%, and 3.8% of the DALYs, respectively [21]. However, these results were based on modelled instead of individually assessed data. Besides, it was not possible to combine health behaviours to investigate whether a combination of health behaviours is directly associated to DALYs in a dose–response manner. The association between several healthy behaviours combined and DALYs has not yet been studied.

We recently showed that DALYs can be used at an individual level in a prospective cohort study with observed instead of modelled data to study relations of risk factors with total disease burden, while accounting for confounding factors [22]. Using this approach, we aim to investigate the combined impact of several modifiable health behaviours, i.e., physical activity, smoking, diet, and BMI, on DALYs by combining these behaviours into a simple health behaviour score using data from the EPIC-NL study.

## Methods

### EPIC-NL study

The EPIC-NL study consists of the two Dutch contributions to the European Prospective Investigation into Cancer and Nutrition (EPIC), which were set up simultaneously between 1993 and 1997. The design and rationale of the EPIC-NL study has been described in detail elsewhere [23]. In brief, the Prospect-EPIC study includes 17,357 women aged 49 to 70 years living in Utrecht and vicinity who participated in the nationwide Dutch breast cancer screening programme. The MORGEN-EPIC cohort consists of 22,654 men and women aged 20 to 65 years selected from random samples of the Dutch population in three different towns (Doetinchem, Amsterdam, and Maastricht). At baseline, a general questionnaire and a food-frequency questionnaire were administered and a physical examination was performed. This study was conducted according to the guidelines laid down in the Declaration of Helsinki and all procedures involving human subjects were approved by the institutional review board of the University Medical Center Utrecht (Prospect) and the Medical Ethical Committee of TNO Nutrition and Food Research (MORGEN). Written informed consent was obtained from all subjects.

From the total cohort (n = 40,011), subjects who did not give permission for linkage with disease registries were excluded (n = 2,910). Furthermore, men and women who suffered from any of the studied diseases (cancer, coronary heart disease (CHD), cerebrovascular accident (CVA), diabetes mellitus, chronic obstructive pulmonary disease (COPD), asthma, Parkinson’s disease, rheumatoid arthritis, osteoarthritis, and inflammatory bowel disease (IBD)) at baseline (n = 3,583) were excluded. Additionally, we excluded subjects without information on dietary intake (n = 142) or with implausible high or low scores for total energy intake (those in the top 0.5% and bottom 0.5% of the ratio of reported energy intake over estimated energy requirement based on basal metabolic rate) (n = 310). The final study population consisted of 33,066 men and women.

### Measurements

The general questionnaire included questions on demographics, presence of chronic diseases, and risk factors for chronic diseases, such as smoking behaviour and level of education (categorized as low (primary education up to those completing advanced elementary education), average (intermediate vocational education and higher general secondary education), or high (higher vocational education and university)). BMI was calculated from height and weight, which were measured during the physical examination. Daily dietary intakes were obtained from a self-administered validated food frequency questionnaire containing questions on the usual frequency of consumption of 79 main food items during the year preceding enrolment. This questionnaire allowed the estimation of the average daily consumption of 178 foods. Physical activity was assessed using the EPIC physical activity questionnaire and categorized according to the validated Cambridge Physical Activity Index (CPAI) [24]. This four-category index (inactive, moderately inactive, moderately active, or active) was derived by cross-classifying three questions referring to activities during the last year against classification of work activity. Because there was no information on physical activity (14%), smoking status (0.4%), educational level (0.8%), alcohol intake (categories) (3.3%), and/or BMI (0.1%) for some of the participants, missing data was imputed using single linear regression modelling (SPSS MVA procedure).

### Health behaviour score

We investigated four important lifestyle factors combined into a previously defined pragmatic health behaviour score [25]. These lifestyle factors, all assessed at baseline, are smoking status, physical activity, BMI, and diet.

For smoking status, participants were grouped in two categories: (0) ever smokers (current and former smokers) and (1) never smokers. BMI was categorized in (1) BMI <25 (normal weight) and (0) BMI of 25 or higher (overweight and obese). Although being overweight or obese is not a lifestyle factor per se, it is a commonly included component in lifestyle indices beside physical activity, smoking, and dietary intake. For physical activity, the CPAI index was dichotomised into (0) inactive and (1) active (moderately inactive, moderately active, or active). The fourth lifestyle factor, diet, was categorized by adherence to the Mediterranean diet that has previously shown a beneficial impact on disease burden [26-29]. Adherence was measured using the modified Mediterranean Diet Score (mMDS) that consists of 8 components (fruit, nuts and seeds, vegetables, legumes, fish, cereals, unsaturated to saturated fat ratio, meat (products), and dairy) after excluding the alcohol component. For each component, 1 point was allocated depending on whether the intake was above or below the population median. Subsequently, these points were summed into one score that ranges from 0 (low adherence) to 8 (high adherence). For the health behaviour score, this mMDS score was further dichotomized into (0) low adherence (0 to 4 points) and (1) high adherence (5 to 8 points). We did not include alcohol in our health behaviour score. A moderate alcohol intake is associated to lower CVD risk but on the other hand related to a higher risk of breast cancer. Furthermore, excessive alcohol intake causes many health-related harms [30]. Therefore, we decided not to include moderate alcohol consumption as a potential beneficial behaviour and instead adjusted for alcohol consumption in the analyses.

We constructed the total health behaviour score by assigning 1 point per healthy behaviour (never smoking, normal weight, physically active, high adherence to Mediterranean diet), creating a total health behaviour score ranging from 0 to 4 points.

### Endpoint assessment

Participants were followed for mortality and morbidity through linkage with several registries. The selection of the diseases was based on their prevalence and disease burden in the Netherlands, but also on the availability of data information sources. Information on vital status and the date of death was obtained through linkage with municipal registries. The cause of death was obtained from Statistics Netherlands. Information on disease occurrence (cancer, CHD, CVA, diabetes mellitus, COPD, asthma, Parkinson’s disease, rheumatoid arthritis, osteoarthritis, and IBD) was obtained from the National Cancer Registry and the national hospital discharge diagnosis database from the Dutch National Medical Registry. The National Cancer Registry provided information on the type of cancer and the date of histological diagnosis. The national hospital discharge diagnosis database provided the date of diagnosis for CHD, CVA, diabetes mellitus, COPD, asthma, Parkinson’s disease, rheumatoid arthritis, osteoarthritis, and IBD. First diagnosis of the disease was assumed to be at the date of hospital discharge. The national hospital discharge diagnosis database was linked to the cohort with a validated probabilistic method using the following information: date of birth, gender, postal code, and name of the general practitioner [31]. Self-report and urinary glucose strip tests (Prospect-EPIC) provided additional information on diabetes mellitus at study entry. New diabetes mellitus cases ascertained during follow-up were verified against information of the general practitioner or pharmacist [32]. Follow-up was complete until 31 December 2007.

### Computation of DALYs

**Figure 1:**
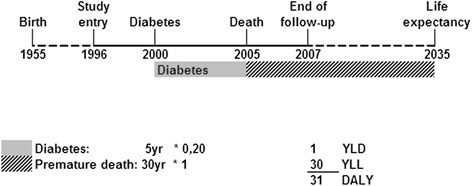
**Example of DALY calculation.** Reproduced from Struijk et al. [22].

DALYs, i.e., the sum of the Years Lost due to Disability (YLD) and the Years of Life Lost due to premature mortality (YLL) [33], were calculated for each individual in the cohort. The YLL are computed as the number of years death occurred earlier than expected. The expected number of life years are the remaining years that a person of a certain age is expected to live on average defined at time of death, loss of follow-up, or end of follow-up [22]. The expected life expectancy was obtained from statistics Netherlands, which provides age, sex, and calendar year-specific life expectancies based on mortality rates [34]. The YLD are calculated by the number of years a person lives with a disability multiplied by a disability weight reflecting the severity of that disability. The disability weights were derived from the Dutch Disability Weight study by three panels of medical experts that evaluated a large number of disease stages using techniques such as person trade-off [35]. The disability weights can range between 0 (no burden) and 1 (death) (Additional file 1: Table S1). The years lived with a chronic disease are calculated from the disease onset until death or until the end of life expectancy. One DALY represents the loss of 1 year in full health. For example, a person who lived 5 years with diabetes (disability weight 0.20) and died 30 years before his life expectancy obtains 1 YLD and 30 YLL, which equals 31 healthy years of life lost (31 DALYs; Figure 1). The calculation of individual DALYs in a cohort and the advantages and disadvantage of different ways of doing that, have been described in more detail elsewhere [22].

### Statistical analysis

Due to the distribution of the DALYs, i.e., a peak at 0 and a normal distribution in participants with DALYs >0, we used a two-part model to estimate the association of the individual lifestyle factors and the health behaviour score with DALYs as dependent variable [36]. This two-part model combines the estimation of the probability of having DALYs using logistic regression with the estimation of the number of DALYs among participants with DALYs >0 using linear regression. Confidence intervals were constructed with bootstrapping (500 samples). The individual health behaviours smoking status (never, former, and ever smokers), physical activity (CPAI index), BMI (normal weight (<25), overweight (25–30), obese (≥30)), and diet (mMDS score 0–2, 3–5, 6–8) were analyzed categorically. The health behaviour score was analyzed categorically using the health behaviour score 0 as the reference. The *P* for trend was estimated by applying a linear regression to the estimates of the categories. The analyses were adjusted for age, sex, education level, alcohol intake (ethanol in g/day and a categorical alcohol variable (never, quit, <1 drink/week, current drinker)), and energy intake. For the individual lifestyle factors, additional adjustment was done for smoking status and intensity (categorized as never, former (quit smoking >20 years ago, quit 10–20 years ago, quit ≤10 years ago), current smoker (1–15 cigarettes/day, 16–25 cigarettes/day, >25 cigarettes/day, pipe or cigar smoker), BMI (continuous), physical activity (CPAI index), and diet (continuous mMDS score) when the covariate was not the variable of interest. We investigated interaction with age and sex by including an interaction term in the logistic and linear model. We conducted additional analyses to investigate the effect of stratifying the analysis between the health behaviour score and DALYs for sex and age (>50/≤50 years), and restricting to several high-risk subgroups, persons with one of the four risk factors, hypertension or high cholesterol (total cholesterol/HDL ratio above 5)). Furthermore, we run analysis replacing BMI with waist circumference (1 point if waist circumference is below 94 cm for males or below 80 cm for females), and including a moderate alcohol intake (10–50 g of alcohol a day for men and 5–25 g of alcohol a day for women) in the mMDS score and without adjusting the analysis for alcohol intake.

Statistical analyses were conducted using SAS 9.2 (SAS Institute, Cary, US), except imputation which has been conducted using SPSS 14.0 (Chicago, IL, USA).

## Results

Baseline characteristics of the study population by the health behaviour score are shown in Table 1. Participants with a higher health behaviour score were young, female, and highly educated (Table 1). After a median follow-up of 12.4 years, 6,647 disease incidences and 1,482 deaths were documented. During the entire follow-up period, 68,225 healthy years of life were lost (68,225 DALYs).

**Table 1:** **Baseline characteristics of 33,066 EPIC-NL participants by health behaviour score**

	**Health behaviour score**				
**Score**	**0**	**1**	**2**	**3**	**4**
	**n = 621**	**n = 7,192**	**n = 13,824**	**n = 9,215**	**n = 2,214**
Gender, % male	36.7	31.2	25.0	23.4	22.8
Age (years)	53.0 ± 9.4	50.8 ± 10.3	49.3 ± 11.7	47.1 ± 12.7	45.2 ± 13.3
Education					
High	11.4	14.0	18.1	27.5	34.6
Middle	33.7	38.1	40.3	42.5	42.8
Low	54.9	48.0	41.6	30.1	22.6
Smoking status					
Never	0.0	4.7	33.6	19.6	100
Former	41.6	49.3	32.5	62.3	0.0
Current	58.5	46.0	34.0	18.1	0.0
Physical activity					
Active	0.0	36.4	43.7	46.5	50.1
Moderately active	0.0	23.2	27.2	29.1	27.1
Moderately inactive	0.0	25.7	25.4	23.5	22.9
Inactive	100	14.7	3.7	1.0	0.0
BMI (kg/m^2^)	29.1 ± 3.7	28.0 ± 3.5	25.8 ± 3.9	23.9 ± 3.3	22.4 ± 1.6
Waist (cm)	95.7 ± 10.7	91.6 ± 10.8	85.3 ± 10.9	80.4 ± 9.6	76.6 ± 6.9
mMDS score	3,0 ± 1,0	3.1 ± 1.0	3.8 ± 1.4	4.7 ± 1.4	5.6 ± 0.8
Energy intake (g/day)	1976 ± 584	2044 ± 609	2057 ± 614	2082 ± 610	2113 ± 590
Ethanol (g/day)	14.5 ± 20.2	13.3 ± 18.0	11.2 ± 15.3	9.8 ± 13.0	7.5 ± 10.1

Baseline characteristics are presented as mean ± SD or %. mMDS, Modified Mediterranean Diet Score.

Table 2 shows the associations of each individual health behaviour with DALYs. All four health behaviours were associated with a significantly lower disease burden both in crude and adjusted analyses. These associations were strongest for smoking and BMI. Both never-smokers and normal weight persons lived approximately a year longer in good health than current smokers (mean DALYs: −1.15; 95% CI: −1.30 to −0.99) and obese persons (mean DALYs: −1.04; 95% CI: −1.23 to −0.86), respectively. Physically active persons lived approximately 6 months longer in good health than physically inactive persons (mean DALYs: −0.54; 95% CI: −0.80 to −0.27). The association was weakest for diet but still significant with approximately 3 months longer life in good health for persons with a high adherence to a Mediterranean diet compared to those with a low adherence (mean DALYs: −0.24; 95% CI: −0.44 and −0.02).

**Table 2:** **Regression coefficient and 95% confidence intervals (CI) of the association between lifestyle factors and DALYs among 33,066 EPIC-NL participants**

	**Lifestyle categories**				***P*** **trend**
**Smoking status**	**Current**	**Former**	**Never**		
n	10,035	10,251	12,780		
DALY, mean ± SD	2.59 ± 6.10	1.98 ± 4.77	1.71 ± 4.53		
Crude	Reference	−0.61 (−0.76 to −0.46)	−0.88 (−1.01 to −0.73)		<0.01
Adjusted model	Reference	−0.91 (−1.07 to −0.75)	−1.15 (−1.30 to −0.99)		<0.01
**BMI category**	**BMI >30**	**BMI 25–30**	**BMI <25**		
n	4,372	13,142	15,552		
DALY, mean ± SD	3.02 ± 5.59	2.28 ± 5.20	1.61 ± 4.89		
Crude	Reference	−0.74 (−0.91 to −0.55)	−1.41 (−1.58 to −1.23)		<0.01
Adjusted model	Reference	−0.69 (−0.85 to −0.50)	−1.04 (−1.23 to −0.86)		<0.01
**mMDS score**	**Low (0–2)**	**Moderate (3–5)**	**High (6–8)**		
n	5,159	22,673	5,234		
DALY, mean ± SD	2.28 ± 5.49	2.07 ± 5.13	1.80 ± 4.80		
Crude	Reference	−0.21 (−0.40 to −0.05)	−0.48 (−0.67 to −0.29)		<0.01
Adjusted model	Reference	−0.10 (−0.27 to 0.06)	−0.24 (−0.44 to −0.02)		0.02
**Physical activity**	**Inactive**	**Mod. inactive**	**Mod. active**	**Active**	
n	2,273	8,038	8,696	14,059	
DALY, mean ± SD	3.11 ± 6.13	2.15 ± 5.09	2.01 ± 5.10	1.88 ± 4.99	
Crude	Reference	−0.96 (−1.21 to −0.66)	−1.09 (−1.36 to −0.82)	−1.23 (−1.47 to −0.95)	<0.01
Adjusted model	Reference	−0.53 (−0.78 to −0.25)	−0.49 (−0.75 to −0.23)	−0.54 (−0.80 to −0.27)	<0.01

Multivariate model: adjusted for gender, age at recruitment, education level, energy intake, ethanol g/day, and drinking alcohol (never, quit, <1 drink/week, yes), and adjusted for the other individual lifestyle factors smoke duration and intensity, BMI (continuous), CPAI (4 cat), mMDS (continuous).

Combining these health behaviours into a simple health behaviour score showed that persons with all four health behaviours lived approximately 2 years longer in good health compared with those without any of the health behaviours (mean DALYs: −2.13; 95% CI: −2.65; −1.62; Table 3). The difference between one and no health behaviours was largest with almost a life year lost in good health (mean DALYs: −0.89; 95% CI: −1.39 to −0.39).

**Table 3:** **Regression coefficient and 95% CI of the association between health behaviour score and DALYs among 33,066 EPIC-NL participants**

	**Health behaviour score**					***P*** **trend**
**Score**	**0**	**1**	**2**	**3**	**4**	
n	621	7,192	13,824	9,215	2,214	
DALY, mean ± SD	4.04 ± 6.86	2.75 ± 5.74	2.09 ± 5.10	1.57 ± 4.67	1.16 ± 4.04	
Crude	Reference	−1.28 (−1.83 to −0.72)	−1.95 (−2.48 to −1.36)	−1.92 (−2.45 to −1.36)	−2.46 (−2.99 to −1.87)	<0.01
Multivariable model	Reference	−0.89 (−1.39 to −0.39)	−1.39 (−1.85 to −0.90)	−1.75 (−2.23 to −1.24)	−2.13 (−2.65 to −1.62)	<0.01

Multivariable model: adjusted for gender, age at recruitment, education level, energy intake, ethanol g/day and drinking alcohol (never, quit, <1 drink/week, yes).

Interaction terms between the health behaviour score or lifestyle factors and age or sex were not statistically significant (*P* >0.05). The associations for persons with all four health behaviours compared to those without any were similar for men and women (mean DALYs men: −2.02; 95% CI: −2.97 to −1.15; mean DALYs women −2.14; 95% CI: −2.73 to −1.46), as well as for younger and older participants (≤50 years mean DALYs: −2.11; 95% CI: −3.33 to −0.75; >50 years mean DALYs −2.07; 95% CI: −2.68 to −1.50) (data not shown in table). Restricting the analysis to individuals with one of the four risk factors yields comparable results. The associations between the health behaviour score and DALYs when restricting the analyses to persons with hypertension are slightly stronger (4 vs. 0 health behaviours, mean DALY: −2.51; 95% CI: −3.31 to −1.75). Analysis among persons with high cholesterol shows that participants with all four health behaviours live on average 3.72 (95% CI: −4.75 to −2.77) years longer in good health compared to those without the four health behaviours. When replacing BMI by waist circumference, we observed similar results with two more life years in good health for all four health behaviours compared to no health behaviours (mean DALYs: −2.07; 95% CI: −2.59 to −1.57) (data not shown in table). Adding the alcohol component to the mMDS did not materially change the findings (mean DALYs 4 vs. 0 health behaviours: −2.25; 95% CI: −2.80 to −1.73) (data not shown in table).

## Discussion

Our prospective study showed that adhering to a healthy lifestyle such as non-smoking, maintaining a low BMI, being physically active, or consuming a healthy diet, results in a lower disease burden. Persons who adhere to all four healthy lifestyle factors lived a minimum of 2 years longer in good health. Increasing from 0 to 1 health behaviour on average resulted in an additional healthy life expectancy of 10.5 months, while each additional increase in health behaviour resulted in an approximately 5 months further improvement in health.

Thus far, DALYs were mainly used in global burden of disease studies, which aim to define the health status in (different parts of) the world and over time, using population data and modelling. The latest Global Burden of Disease study from 2010 investigated the DALYs attributable to unhealthy lifestyle [21] and the results are to a large part in line with the results from our study. In our study, tobacco smoking was responsible for the highest disease burden followed by overweight/obesity, physical inactivity, and an unhealthy diet. In the Global Burden of Disease study, tobacco smoking was the leading modifiable lifestyle factor, accounting for 6.3% of the disease burden. This is followed by a diet low in fruits that accounted for 4.2% of the global DALYs, a high BMI (3.8% of global DALYS), and physical inactivity (2.8% of global DALYs). The reason for the large impact of diets low in fruit in the global disease burden is the high exposure to low fruit diets, as in many regions a diet low in fruit is common. DALY estimates in global burden of disease studies represent disease burden at the population level and depend on the prevalence of the exposure. A high exposure of unhealthy behaviour then automatically results in a larger disease burden. In our study, we are able to look at the association between an unhealthy lifestyle behaviour and disease burden in more detail.

The individual lifestyle variables are not independent. The analyses of physical activity in association with disease burden are adjusted for BMI, which decreases the size of the estimate. The results for the individual lifestyle factor physical activity showed that compared to being inactive, the estimate for being moderately inactive is similar to that of being active. This indicates that it is mainly important not to be inactive and that level of activity is less important. However, it may also reflect misclassification between the moderately active and moderately inactive categories. Furthermore, it has to be taken into account that differences in results of the individual lifestyle factors may partly be caused by the categorization used.

Our study results show that persons with a healthy lifestyle live longer in good health. This is in line with several studies that found an inverse association between a healthy lifestyle and the incidence of stroke [6-8], type 2 diabetes [9,10], colorectal cancer [11], and mortality [12-18]. A study among participants from EPIC-Potsdam investigated the impact of lifestyle on developing any of the major chronic diseases (CHD, stroke, diabetes, cancer) [19]. They also found a large beneficial impact on chronic disease risk for participants with all four health behaviours (never smoking, BMI <30, 3.5 h/wk or more of physical activity, and adherence to a healthy diet) with a hazard ratio of 0.22 (95% CI: 0.17 to 0.28) compared to unhealthy lifestyle. Similarly, they also found a clear inverse association for each additional health behaviour independent of the number of health behaviours a person already had. There are a few studies that used another summary health measure as an outcome. A study among persons from EPIC-Norfolk found that the mean number of quality-adjusted life years (QALY) were higher among those with more health behaviours [37]. In this study, no regression analysis or adjustment for confounders was done and, in addition, a constant quality of life was assumed during the whole follow-up period. A reduced quality-adjusted life expectancy was found for persons with unhealthy behaviours in a Danish study [38]. Direct comparison of the estimates is not possible because in the Danish study a life table approach was used to calculate the life expectancy of persons with a specific risk factor combined with QALY utility weights.

The main strength of our study is that we use a prospective cohort study with a summary health measure as an outcome. The association between lifestyle factors and overall health is most likely of greater interest to the general population than risk of a specific disease or mortality from a specific disease. Furthermore, DALYs take into account the severity of and the time lived with a specific disease combined with premature mortality. In contrast to global and national burden of disease studies, the use of the DALYs in a large prospective cohort has the benefit that individual data is used, which enables the use of direct association methods between one or a combination of lifestyle factors and DALYs and direct adjustment for confounders.

However, several limitations need to be addressed. Our estimates underestimate the true association due to the left and right truncation of the cohort [22]. Participants who were still alive at the end of follow-up were assumed to stay in the same state of health until their expected age of death. In reality, part of the participants who were still disease-free at the end of follow-up (December 2007) will develop diseases before eventually dying. This is not accounted for in the current analysis. For those living with a disease at the end of follow-up, DALYs may be underestimated as well, since their life expectancy is assumed to be similar to that of a healthy person, while they are more likely to die earlier. Additionally, due to the relatively healthy cohort and exclusion of participants with prevalent diseases at baseline, many participants, including those with an unhealthy lifestyle, were still disease-free at the end of follow-up. Our results, based on an observation time of 12 years in healthy participants, are therefore a minimum estimation of the true impact of lifestyle on disease burden. Full impact of a combination of lifestyle factors on healthy life years can only be observed after longer follow-up, preferably until complete extinction of the cohort. An additional reason for underestimation of the association is that we were not able to include all diseases such as Alzheimer’s disease, depression, and infectious diseases. Furthermore, the incidence of some of the diseases that were included is probably underestimated because our data were largely based on hospital discharge diagnoses. Not all relevant diseases could be incorporated in our analysis and for other major diseases only severe cases resulting in hospitalization were included. In addition, the registered date of onset of disease (date of hospital discharge) is likely to be later than the true date of onset when the disease started to contribute to disease burden. However, we did include those diseases that are most strongly associated with lifestyle. Unfortunately, no information was available to expand the health behaviour score with additional health behaviours such as sleep, weak social support, and screen use. Another limitation is that we cannot rule out the possibility of residual confounding due to misclassification of any of the lifestyle behaviours.

## Conclusions

Our study clearly shows the benefits of a healthy lifestyle. The disease burden among persons who never smoked, maintain a normal BMI, are not physically inactive, and adhere to a healthy diet is considerably lower than that of those who do not adhere to any of the healthy lifestyle behaviours and results in a minimum of 2 years longer life in good health. Each additional healthy lifestyle factor contributes significantly to a longer life in good health independent of the lifestyle score someone already has.

## Additional file

Additional file 1: Table S1. Disability weights for disabilities and different cancer types.

## Abbreviations

BMI: Body mass index
CHD: Coronary heart disease
CI: Confidence interval
COPD: Chronic obstructive pulmonary disease
CPAI: Cambridge Physical Activity Index
CVA: Cerebrovascular accident
DALYs: Disability-Adjusted Life Years
EPIC: European Prospective Investigation into Cancer and nutrition
IBD: Inflammatory bowel disease
mMDS: Modified Mediterranean diet score
QALY: Quality-Adjusted Life Years
SD: Standard deviation
YLD: Years Lost due to Disability
YLL: Years of Life Lost
